# Operant Up-Conditioning of the Tibialis Anterior Motor-Evoked Potential in Multiple Sclerosis: Feasibility Case Studies

**DOI:** 10.1155/2018/4725393

**Published:** 2018-07-15

**Authors:** Aiko K. Thompson, Briana M. Favale, Jacqueline Velez, Patricia Falivena

**Affiliations:** ^1^Department of Health Sciences and Research, College of Health Professions, Medical University of South Carolina, Charleston, SC 29425, USA; ^2^Helen Hayes Hospital, New York State Department of Health, West Haverstraw, New York City, NY 10993, USA

## Abstract

Damage to the corticospinal pathway often results in weak dorsiflexion of the ankle, thereby limiting the mobility of people with multiple sclerosis (MS). Thus, strengthening corticospinal connectivity may improve locomotion. Here, we investigated the feasibility of tibialis anterior (TA) motor-evoked potential (MEP) operant conditioning and whether it can enhance corticospinal excitability and alleviate locomotor problems in people with chronic stable MS. The protocol consisted of 6 baseline and 24 up-conditioning sessions over 10 weeks. In all sessions, TA MEPs were elicited at 10% above active threshold while the sitting subject provided 30–35% maximum voluntary contraction (MVC) level of TA background EMG. During baseline sessions, MEPs were simply measured. During conditioning trials of the conditioning sessions, the subject was encouraged to increase MEP and was given immediate feedback indicating whether MEP size was above a criterion. In 3/4 subjects, TA MEP increased 32–75%, MVC increased 28–52%, locomotor EMG modulation improved in multiple leg muscles, and foot drop became less severe. In one of them, MEP and MVC increases were maintained throughout 3 years of extensive follow-up sessions. These initial results support a therapeutic possibility of MEP operant conditioning for improving locomotion in people with MS or other CNS disorders, such as spinal cord injury and stroke.

## 1. Introduction

Transcranial magnetic stimulation (TMS) and its motor-evoked potential (MEP) have been widely used to study corticospinal excitability and connectivity noninvasively [1]. Generally, for a given intensity of stimulation, the larger the response size, the stronger the corticospinal connectivity and/or excitability [1–3]. Corticospinal activity is essential in human motor control, including walking [1, 3–6], and its restoration is important for regaining motor skills after CNS lesions [7–11]. In fact, CNS lesions that damage descending connections from the brain, such as spinal cord injury and multiple sclerosis (MS), reduce MEP size [6, 12–16], reflecting diminished corticospinal function in these populations. In people with MS, particularly, MEP measurements have been found useful in detecting and predicting the progression of disability and recovery [14, 15, 17–21]. Importantly, people with MS can display corticospinal plasticity [22, 23], which plays a key role in motor function recovery [7, 8, 24–28]. Thus, new methods for strengthening corticospinal connectivity and enhancing corticospinal function may improve motor function in people with MS. In the present case studies, we explored the possibility that enhancing the excitability and connectivity of corticospinal pathways to the ankle dorsiflexors through MEP operant conditioning can improve impaired locomotion due to moderately severe secondary progressive MS.

Operant conditioning is a powerful method for modifying a behavior based on its consequences. The past 35 years of spinal reflex operant conditioning studies in monkeys, rats, mice, and humans show that even a simple spinal reflex behavior can be gradually changed through learning and practice [29–31]. Animal studies have revealed that a hierarchy of spinal and supraspinal plasticity (from the cerebellum to sensorimotor cortex to corticospinal tract to spinal cord) is involved in inducing and maintaining the operant conditioning-induced change in the evoked response [31, 32]. Thus, operant conditioning of an EMG-evoked response that induces spinal and supraspinal plasticity may be used as a gateway to encouraging the production of specific patterns of CNS activity that are potentially linked to better motor function recovery after CNS damage [33–36]. In the present studies, an operant up-conditioning protocol was applied to the tibialis anterior (TA) MEP of people with MS who suffer from foot drop. The subjects were trained to strengthen the pathway specifically responsible for generating the TA MEP, so as to improve locomotion that also needs this pathway.

The purpose of this study was to investigate the feasibility and a therapeutic possibility of MEP operant conditioning for improving locomotion in people with chronic stable MS.

## 2. Materials and Methods

### 2.1. MEP Operant Conditioning Protocol

The MEP operant conditioning protocol used in this study is a modified version of the conditioning protocols developed and used in human subjects with and without spinal cord injury [34, 37, 38]. The study protocol was approved by the Institutional Review Board of Helen Hayes Hospital, New York State Department of Health, and all subjects gave written consent prior to participation.

**Figure 1:**
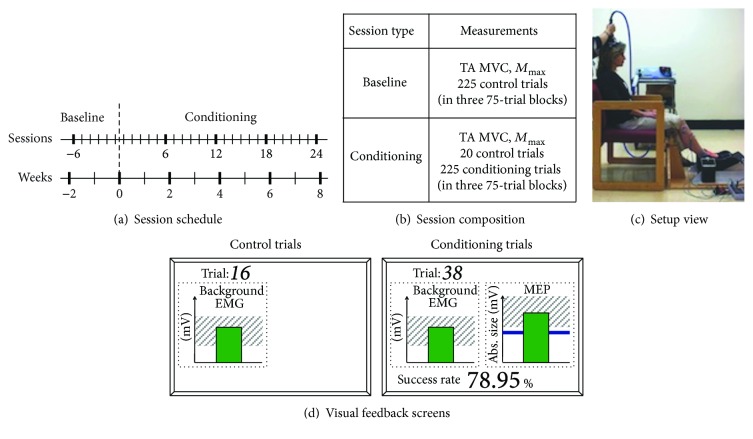
(a) Session schedule. Six baseline sessions are followed by 24 conditioning sessions. (b) Composition of baseline and conditioning sessions. (c) Setup view. The subject sits in a chair with hip, knee, and ankle angles fixed at ≈100°, ≈120°, and ≈110°, respectively, in a custom-made apparatus. (d) Visual feedback screens for control and conditioning trials. In all trials, the number of the current trial within its block is displayed, and the background EMG panel shows the correct range (shaded) and the current value (green vertical bar, updated every 200 ms). If TA EMG activity stays in the correct range for at least 2 s and at least 5.5 s has passed since the last trial, an MEP is elicited. In control trials (left), the MEP panel is not shown. In conditioning trials (right), the shading in the MEP panel indicates the rewarded MEP range for up-conditioning. The dark horizontal line is the average MEP size of the baseline sessions, and the vertical bar is the MEP size (i.e., the average rectified EMG in the MEP interval (e.g., 45–75 ms after TMS)) for the most recent trial (it appears 200 ms after TMS). If that MEP size reaches into the shaded area, the bar is green and the trial is a success. If it falls below the shaded area, the bar is red and the trial is a not a success. The running success rate for the current block is shown at the bottom.

At the beginning of the study, the subject was familiarized with the protocol and TMS in several preliminary sessions. Then, each subject completed 6 baseline sessions and 24 up-conditioning sessions that occurred at a pace of 3 times per week (Figure 1(a)). In one of the subjects, follow-up sessions occurred over the following 3 years at varying intervals (1 week to several months). To avoid variability due to possible diurnal variation in response size [39, 40], a subject's sessions always occurred at the same time of day (i.e., within the same 3 hr time window). In all sessions, TA MEPs were measured while the sitting subject provided a preset level (typically 30–35% maximum voluntary contraction (MVC) level, determined during preliminary sessions) of TA background EMG with ankle, knee, and hip joint angles fixed at ≈100°, ≈120°, and ≈110°, respectively.

At the beginning of each session, self-adhesive surface Ag-AgCl electrodes (2.2 × 3.5 cm, Vermed Inc., Bellows Falls, VT) were placed over the muscle bellies of TA and soleus for EMG recording [13, 41]. EMG signal was amplified, band-pass filtered (10–1000 Hz), and sampled at 5000 Hz. Absolute TA EMG during MVC was measured in two 3 s maximum isometric dorsiflexion trials, while the subject sat in a chair with hip, knee, and ankle joints fixed in a custom-made apparatus (Figure 1(c)). Then, the common peroneal nerve was stimulated at the neck of fibula, using surface electrodes (2.2 × 2.2 cm for cathode, 2.2 × 3.5 cm for anode, Vermed Inc.) and 0.5 ms wide single square pulses delivered from Grass S88 stimulator with an SIU-5 stimulation isolation unit and a CCU1 constant current unit (Astro-Med Inc., West Warwick, RI), while the subject sat at rest. Four EMG responses were averaged at each of 10 stimulus intensities varied from below *M*-wave threshold to just above the maximum *M*-wave (*M*_max_) [42]. This TA *M*_max_ measurement was followed by control MEP trials (Figure 1(b)). In all trials of baseline sessions and the first 20 trials of conditioning sessions, the subject received no feedback as to MEP size (i.e., control MEPs). The subject simply needed to maintain the preset level of TA EMG activity. In contrast, in 225 conditioning trials of each conditioning session (i.e., conditioning MEPs), the subject was encouraged to increase TA MEP size and received immediate feedback as to whether MEP size was above a criterion (i.e., whether the trial was a success, Figure 1(d)). The criterion was based on the average MEP size for the previous block of trials. In each conditioning session, the criterion value for the first block of 75 conditioning trials was determined based on the immediately preceding block of 20 control trials, and the criterion values for the second and third conditioning blocks were based on the immediately preceding block of 75 conditioning trials. The criterion was selected so that if MEP values for the new block were similar to those for the previous block, 50–60% of the trials would be successful [34, 37]. For each block, the subject earned a modest extra monetary reward if the success rate exceeded 50% (see [37] for full details of the protocol.). For all trials, TMS at 10% above threshold was used to evoke the MEP. The same absolute TMS intensity (expressed as % maximum output of the stimulator) was used throughout the study. TA and soleus background EMG levels were kept stable throughout data collection. In order to minimize the session-to-session variability in nerve stimulation and EMG recording, the positions of all electrodes were measured in relation to landmarks on the skin (e.g., scars or moles) during the first preliminary session, and the same measures were used to place the electrodes in all subsequent sessions.

### 2.2. Subjects

Four women (age: 47–56 years) with chronic foot drop due to secondary progressive MS participated. Inclusion criteria were (1) neurologically stable for >6 months; (2) medical clearance to participate (with the expectation that current medication and therapy schedule would be maintained without change for at least 4 months)^∗^; (3) ability to ambulate with or without an assistive device (i.e., Expanded Disability Status Scale (EDSS) 2 to 6 [43]); and (4) clinical signs of weak dorsiflexion of the ankle at least unilaterally. Exclusion criteria were (1) lower motor neuron damage; (2) known cardiac condition; (3) medical instability; (4) cognitive impairment; (5) a history of epileptic seizures; (6) metal implants in the head; and (7) implanted biomedical device in or above the chest. ^∗^Except for subject A, who had been taking 4-aminopyridine (Ampyra®) for 5 years at the time of enrollment, none were on neuromodulatory medication (e.g., baclofen). These subjects maintained their long-term MS medication, such as beta interferons (e.g., Avonex®), rituximab (Rituxan®), and dimethyl fumarate (Tecfidera®), throughout the 10 weeks of main study period.

The subject profiles are summarized in Table 1. In subject B, the MEP could be elicited only in one leg, and therefore that leg was studied. In subjects A, C, and D, the MEP could be elicited in both legs, and the more severely affected leg was studied (i.e., more severe foot drop). Before and after the 24 conditioning sessions, Dr. Falivena and Ms. Velez, who were blinded to the subjects' conditioning results, assessed the clinical and functional states of disability: EDSS [43], evaluation of ankle joint passive range of motion, extent of voluntary movement control with manual muscle testing, and presence of movement abnormalities. 25 ft walking time was measured [44], and a functional independence measure (FIM) motor score was obtained.

### 2.3. Transcranial Magnetic Stimulation

TMS was performed using Magstim 200^2^ and a custom-made double-cone coil (in subjects B, C, and D) or a bat-wing coil (in subject A) with radii of 9 cm (Jali Medical Inc., Woburn, MA), held over the scalp such that the induced current flowed in the posterior–anterior direction in the brain. When the absolute TA EMG level was maintained within the preset range (determined as 30–35% MVC level during preliminary sessions, and not changed throughout the entire study [34, 37]), an MEP was elicited by TMS, with a minimum interval of >5.5 s between stimuli. In preliminary sessions, the optimal TMS location at which the lowest stimulus intensity elicited the TA MEP was determined by moving the coil over the scalp. At the optimal location, the input-output curve of the corticospinal pathway was measured by increasing the TMS intensity, represented as a percentage of the maximum current that can be discharged into the coil, in steps of 5% until the MEP reached its plateau. Four MEPs were collected at each intensity, and the peak-to-peak and mean rectified amplitudes were plotted against the stimulus intensity [13, 45–47]. From the input-output curve measurement in several preliminary sessions, a stimulus intensity that was ≈10% above threshold was determined for each subject, and the same absolute TMS intensity was used throughout all the baseline and conditioning sessions.

Along with control and conditioned MEP size measurements, we also measured the silent period (SP) as the period from the end of the MEP to the recovery of background EMG activity in 50% of the responses [13, 45, 46, 48].

### 2.4. Locomotor EMG Measurement

Overground locomotor EMG activity was measured before and after the 24 conditioning sessions. Subject B, who wore an ankle foot orthosis on her unconditioned leg, was asked to remove it for this measurement. Surface EMG was recorded from the TA, soleus, biceps femoris, and vastus lateralis bilaterally. To detect foot contact for each leg, foot-switch cells were inserted between the subject's shoes and feet. For analysis, the foot-contact signal was used to define the beginning of the step cycle. Rectified EMG signals from individual steps were normalized to the mean step cycle time and averaged together to obtain the locomotor EMG activity over the step cycle [34, 49, 50]. Fifty or more steps were averaged. For quantitative analysis, the step cycle was divided into 12 equal bins and EMG activity was averaged for each bin. Then, each bin's average EMG amplitude was expressed in percent of the amplitude in the bin with the highest amplitude [38]. To determine for each muscle the extent of EMG modulation over the step cycle, a modulation index (MI) was calculated in percent as 100 × [(highest bin amplitude − lowest bin amplitude)/highest bin amplitude] [34, 38]. In each muscle, the MIs across the 12 bins were compared before and after conditioning.

### 2.5. Data Analysis

For each session of each subject, we calculated average MEP sizes for the 20 within-session control trials and for all three 75-trial blocks together. For these calculations, MEP size was defined as average absolute EMG in the MEP interval minus average background EMG. Changes in these MEP sizes across sessions were quantified in percent of their average values for the 6 baseline sessions. We also determined for each subject the final effects of conditioning on the conditioned MEP by averaging the MEPs for the 75-trial conditioning blocks of conditioning sessions 22–24 and expressing the result in % of the average MEP for the 75-trial blocks of the 6 baseline sessions. (Thus, a value of 100% indicates no change in the MEP.) The final effect on the control MEP was calculated by averaging the MEPs for the 20 within-session control trials of conditioning sessions 22–24 and expressing the result in % of the average MEP for the first 20 trials of the 6 baseline sessions (see [37]).

To determine for each subject whether MEP up-conditioning was successful, the average conditioned MEPs of the final 6 conditioning sessions were compared to the average MEPs of the 6 baseline sessions by unpaired *t*-test (two-tailed) [34, 37]. To evaluate in each subject the stability of the *M*_max_ and background EMG levels over all sessions and assess changes in MVC and SP, a factorial ANOVA was used across successive 6-session blocks (i.e., baseline sessions 1–6 and conditioning sessions 1–6, 7–12, 13–18, and 19–24) [37]. TA *M*_max_ and TA and soleus background EMG levels in control and conditioning trials remained stable throughout the study (*p* > 0.07 for all parameters by one-way factorial ANOVA in each of the subjects). Typically, TA *M*_max_ remained within ±15% to the mean across all the sessions, TA background EMG remained within ±5%, and soleus background EMG remained under 5 *μ*V (i.e., resting EMG level) throughout the study.

## 3. Results

In 3 of the 4 subjects, TA MEP increased significantly, together with the TA MVC; and at the end of conditioning, all three spontaneously reported better leg movement in their daily walking. Here, we describe each subject's results individually.

### 3.1. Subject A

**Figure 2:**
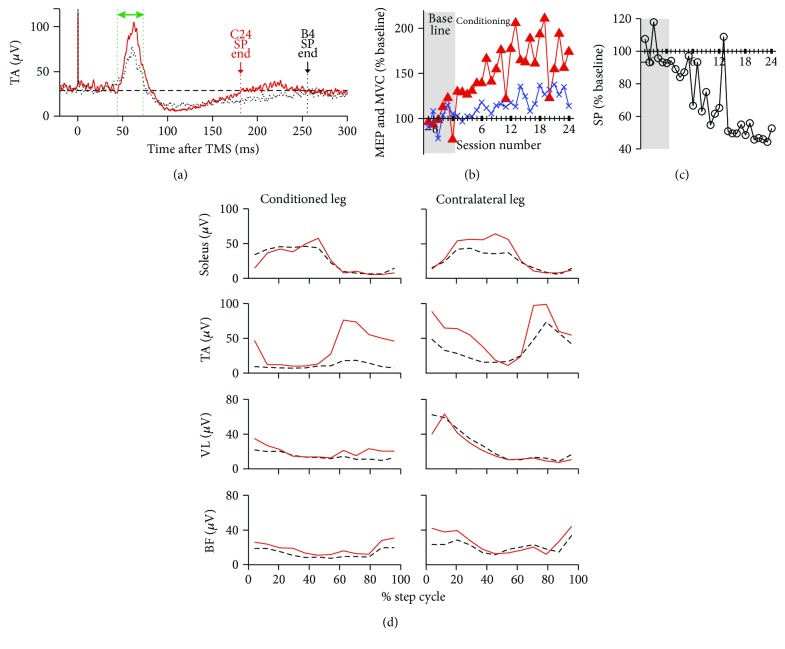
Changes in tibialis anterior (TA) motor-evoked potential (MEP), maximum voluntary contraction (MVC), and silent period (SP) over the course of study, and bilateral locomotor EMG activity in subject A. (a) Rectified EMG signals from the fourth baseline session (dashed black) and the last (i.e., 24th) conditioning session (solid red). Two hundred twenty-five responses were averaged together for each sweep. A pair of dashed vertical lines (green) indicates the time window for this subject's MEP size calculation. The horizontal line at 30 *μ*V indicates the background EMG level. (b) Mean MEP size (i.e., the mean of 225 control (in baseline sessions) or 225 conditioned (in conditioning sessions) MEP trials) (filled triangle) and MVC (cross) in 6 baseline (shaded part of the panel) and 24 conditioning sessions. (c) Mean SP duration (i.e., as marked in panel (a)). (d) Rectified locomotor EMG activity in soleus, TA, vastus lateralis (VL), and biceps femoris (BF) muscles of both legs before (dashed black) and after (solid red) conditioning. The step cycle is divided into 12 equal bins, starting from foot contact. Generally, bins 1–7 (i.e., up to 60% of the step cycle) are for the stance phase and bins 8–12 (i.e., 60–100% of the step cycle) are for the swing phase. After successful MEP up-conditioning, EMG modulation over the step cycle becomes greater in the muscles of both legs.

Over the course of conditioning, her left TA MEP increased progressively. Her final conditioned MEP size was 175% of the baseline value (Figure 2(a)). This increase consisted of a 33% increase in the control MEP (reflecting long-term across-session change) and a 42% within-session increase (i.e., task-dependent change) (not shown in figures, see [34, 35, 37] for discussion of them). TA MVC also increased gradually; the final MVC was 128% of the baseline value (Figure 2(b)), while the SP duration decreased steadily from 205 ms in baseline sessions to 98 ms in the conditioning sessions 22–24 (Figure 2(c)).

There were several noteworthy observations over the course of study. First, this subject had no visible voluntary ankle dorsiflexion at the beginning and needed a cane to compensate for the limited motion in the left leg. After 24 conditioning sessions, she was able to flex the left ankle even against resistance (Table 1); and at the post conditioning clinical evaluation, she surprised Dr. Falivena by saying that she forgot to bring a cane. She commented that her cane usage had decreased over the course of study. After conditioning, 25 ft walking time was decreased, indicating a 28% increase in walking speed (Table 1).  Figure 2(d) shows the changes in locomotor EMG activity after conditioning. The conditioned leg's TA burst during the swing phase is clearly increased, enabling ankle dorsiflexion during the swing phase and heel contact at the beginning of the stance phase. Thus, after conditioning, this subject no longer suffered from foot drop. As Figure 2(d) also shows, the swing phase burst was increased in the contralateral TA as well. Locomotor EMG MI was increased in 6 of 8 leg muscles, indicating an overall improvement of EMG modulation during locomotion.

**Figure 3:**
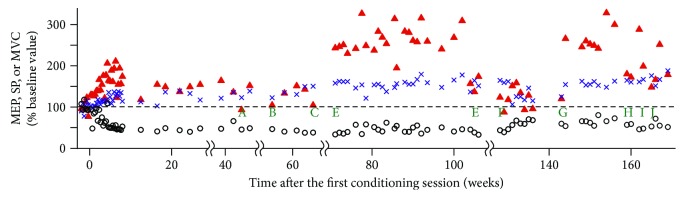
TA MEP (filled triangle), MVC (cross), and SP (circle) over 3.3 years of study including baseline to conditioning to follow-up periods in subject A. During the follow-up period (i.e., after 8 weeks of conditioning), her official diagnosis changed (from secondary progressive MS to relapsing-remitting MS) and so did her medication. Notable events in her life and neurological/medical history during the study period are indicated as A–J. (A) A few days after the first rituximab injection. (B) Feeling tired due to personal life issue. (C) Going through divorce. (D) Cortical mapping had been redone. TMS location was changed from “2.5 cm lateral and 1.5 cm anterior to the vertex”. (E) Kidney infection. (F) MS relapse diagnosed; started taking dimethyl fumarate (tecfidera); her gait had worsened. No visible ankle dorsiflexion. (^∗^Visible dorsiflexion returned several weeks later.) (G) Frequent urinary tract infection for the past several weeks. (H) Started taking ropinirole hydrochloride (requip). Also started on tamsulosin and oxybutynin for UTI treatment/prevention. (I) Her mother just had a stroke. (J) Started taking interferon beta Ia (avonex). Throughout this lengthy follow-up and many life events, the MEP and MVC increases and the SP decrease associated with MEP up-conditioning persisted.

In this subject, 66 follow-up sessions were performed over 161 weeks (3 years) after the last conditioning session (Figure 3). During this period, her official diagnosis changed from secondary progressive MS to relapsing-remitting MS, and the medication changed accordingly. Also, on week 70, we repeated cortical mapping of the TA MEP. At that point, we found that the MEP size at the vertex was similar to that elicited at the original optimum location (i.e., 2.5 cm lateral and 1.5 cm anterior to the vertex). Thus, from week 71 on, TMS was applied at the vertex, not at the original optimum location. Also, the TMS intensity and coil were changed from 80% of stimulator's maximum output with a bat-wing coil to 70% with a double-cone coil, which generated control MEPs comparable to those generated by 80% with the bat-wing coil. Larger conditioned MEP size observed from week 71 on may be partially due to less-focused wider-spread stimulation with the double-cone coil. Most importantly, over this extensive follow-up, her MVC increase was well maintained (*M*_max_ remained around 3.8 mV for the entire 172 weeks of her study). An increased MEP and decreased SP duration were also present in most of the follow-up sessions. At the study conclusion (i.e., 3 years after conditioning), voluntary ankle dorsiflexion remained visible and improved.

### 3.2. Subject B

**Figure 4:**
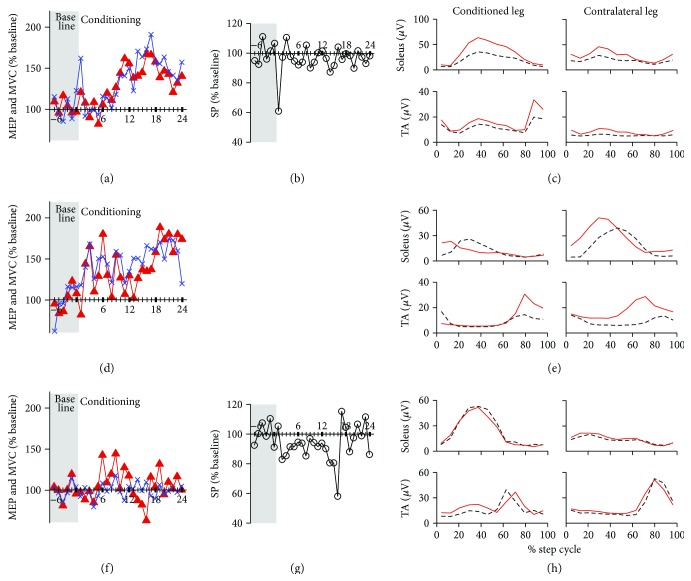
Changes in the TA MEP, MVC, and SP over the course of study and bilateral locomotor EMG in subjects B, C, and D. (a, b, c) Subject B. (a) Mean MEP size (filled triangle) and MVC (cross) in 6 baseline (shaded part of the panel) and 24 conditioning sessions. (b) Mean SP duration. (c) Rectified locomotor EMG activity in soleus and TA of both legs before (black) and after (red) conditioning. After successful MEP up-conditioning, locomotor EMG modulation increases in TA and soleus of both legs; not only the conditioned TA but also the soleus burst amplitude increased. (d, e) Subject C. (d) Mean MEP size and MVC in 6 baseline and 24 conditioning sessions. (e) Rectified locomotor EMG activity in soleus and TA bilaterally, before and after conditioning. After successful MEP up-conditioning, TA EMG burst amplitude increased in both legs. SP duration could not be measured in this subject. (f, g, h) Subject D. (f) Mean MEP size and MVC in 6 baseline and 24 conditioning sessions. (g) Mean SP duration. (h) Rectified locomotor EMG activity in soleus and TA bilaterally, before and after conditioning. In this subject, unlike subjects A–C, MEP up-conditioning had no significant effect.

The TA MEP increased over the course of study (Figure 4(a)). Her final conditioned MEP size was 132% of the baseline value; this came from within-session increase. There was no long-term change in the control MEP. TA MVC increase paralleled MEP change; the final MVC was 142% of the baseline value (Figure 4(a)). SP duration did not change (from 277 ms in baseline sessions to 265 ms in the conditioning sessions 22–24, Figure 4(b)).

After conditioning, the conditioned leg's TA burst in the late swing phase of walking and soleus burst during the stance phase were clearly increased (Figure 4(c)). Locomotor EMG MI was increased in 7 of 8 muscles measured, indicating overall improvement in EMG modulation. These improvements in the locomotor EMG activity, together with improved dorsiflexion strength (Table 1), were associated with a 36% increase in walking speed (Table 1).

### 3.3. Subject C

Her final conditioned MEP size was 172% of the baseline value (Figure 4(d)), which consisted of 45% increase in the control MEP (i.e., long-term increase) and a 27% within-session task-dependent increase (not shown in figures). MVC increase paralleled MEP change; the final MVC was 152% of the baseline value (Figure 4(d)). We were unable to determine the SP duration, as she was unable to maintain the background EMG level after TMS in many of the trials of the baseline sessions.

The TA burst in the swing phase of locomotion increased after conditioning in both the conditioned and the contralateral legs (Figure 4(e)). Locomotor EMG MI was increased in 6 of 8 muscles measured, indicating overall improvement in EMG modulation. However, soon after the last conditioning session, the neurologist noted that her MS resumed to progress; at the post functional assessment that was performed one month after the last conditioning session (this delay of assessment was caused by her physical condition and scheduling conflicts), the walking speed was decreased (Table 1).

### 3.4. Subject D

MEP up-conditioning did not increase her MEP (Figure 4(f)). Her final conditioned MEP size was 106% of the baseline value, which consisted of 9% within-session increase and 3% across-session control MEP decrease. TA MVC did not increase either; the final MVC was 101% of the baseline value (Figure 4(f)). SP duration also did not change over the course of conditioning (234 ms in baseline sessions and 231 ms in the conditioning sessions 22–24, Figure 4(g)). No clear changes were observed in her locomotor EMG activity after conditioning (Figure 4(f)). Locomotor EMG MI was increased in 3 of 8 muscles while unchanged or decreased in 5 of 8 muscles measured, suggesting no overall improvement in EMG modulation. However, her 25 ft walking time was reduced after conditioning by 15% (Table 1). This speed improvement may be a nonspecific (psychological) effect of the procedures (see also [34]). Altogether, the results show that unsuccessful conditioning in this subject was unlikely due to her MS status and did not worsen her gait.

## 4. Discussion

### 4.1. Effects of Up-Conditioning on MEP and SP in People with MS

In three of 4 subjects with MS, up-conditioning successfully increased MEP over the course of 24 conditioning sessions, supporting the possibility and feasibility of MEP operant conditioning in people with chronic stable MS.

In two of the 3 successfully conditioned subjects, the conditioned MEP increase consisted of within-session task-dependent adaptation and cumulative long-term change, similar to previous studies of H-reflex operant conditioning [34, 37, 38]. Since the long-term change represents the plasticity that persists outside of the conditioning paradigm [34, 38, 42], we expected that functional improvement (i.e., in locomotion and MVC) would be linked to the presence and extent of long-term increase in MEP size. However, increased MVC, improved locomotor EMG, and increased walking speed were present even in the subject with little long-term MEP increase (Figures 4(a)–4(c), Table 1). Thus, it is possible that the relationship between the long-term change and functional impact may differ between the pathways targeted by different operant conditioning protocols. Different from reflex conditioning, functional impact of MEP conditioning may be better estimated with the total conditioning-induced change (i.e., the sum of long-term and task-dependent changes) or the task-dependent change of corticospinal excitability. Since corticospinal drive is directly involved in the activation of TA during the swing phase of locomotion [4, 5], after successful MEP up-conditioning, the corticospinal pathways that were trained to increase their connectivity during conditioning trials would also do so during walking.

In all of these subjects with MS, we noticed that the SP duration was very long (i.e., >200 ms) during the baseline sessions. Since baclofen, a common antispastic medication for people with MS, spinal cord injury, and cerebral palsy [51–53], is a GABA_B_ receptor agonist and known to prolong the SP duration [54, 55], one might suspect that the observed long SP would be due to medication. However, that would not be the case here; none of the present subjects had been taking baclofen or any other known antispastic medication (e.g., diazepam). Therefore, it is likely that their long SP reflects their underlying physiology, not GABAergic medication effects. The later part of long SP (e.g., >100 ms) originates from GABA_B_-mediated intracortical inhibition [56–58]. The prolonged SP observed in the present subjects with MS could, thus, be due to enhanced intracortical inhibition [54] that may be linked to their motor impairments [59]. In subject A, the SP duration progressively decreased over the course of study (Figures 2(a) and 2(c)), suggesting a decrease in intracortical inhibition. We have observed similar decreases in SP duration with MEP up-conditioning in neurologically normal subjects, in whom baseline SP duration was not quite long (≈100 ms, [60]). Considering this clear difference in the baseline SP duration between normal subjects and subjects with MS and the fact that SP duration did not decrease in subject B, further studies are needed to investigate the consistency and mechanisms of SP decrease with MEP up-conditioning.

### 4.2. Functional Implications

In 3 of the 4 subjects, operant up-conditioning of TA MEP was successful and was accompanied by increased MVC, increased TA burst during the swing-phase of locomotion, and improved EMG modulation during locomotion. In 2 of the 3 subjects, the ankle dorsiflexor strength and walking speed also improved; in the third subject, disease progression near the end of the study may have prevented walking speed improvement despite significant increases in MEP, MVC, and locomotor EMG modulation. The increased corticospinal drive to the conditioned TA may explain increases in TA MEP, TA MVC, and TA burst amplitude during the swing phase of walking [4, 5, 26], but it cannot explain widespread improvements in locomotor EMG activity; locomotor EMG modulation improved in the ankle extensors, knee flexors, and knee extensors of both legs. This wide effect of MEP up-conditioning is very similar to the locomotor improvements produced by H-reflex down-conditioning in people with SCI [34]. As discussed in detail elsewhere [34, 35], bilateral locomotor EMG improvements in proximal and distal leg muscles probably occurred through additional plasticity in other spinal pathways that are involved in locomotion. The acquisition of a new behavior (e.g., increasing corticospinal excitability for the TA) through 8 weeks of operant conditioning probably triggered a widespread adaptive plasticity in many CNS pathways that resulted in better locomotion [32, 33, 35]. These widespread effects are explainable in terms of the negotiated equilibrium hypothesis [32].

One of the cases presented here (subject A) has an important implication for future therapeutic applications. Over 3 years of follow-up sessions, her TA MEP, MVC, and SP improvements and the voluntary ankle dorsiflexion that she had not had before conditioning were essentially maintained (Figure 3), despite changes in diagnosis, medication, and personal life. This case supports the possibility of long-term maintenance of improvement with periodic follow-up sessions, after completion of initial intense conditioning. The intervals between follow-up sessions varied from a week to months in this subject; the minimum effective follow-up interval needs to be determined through thorough systematic studies.

#### 4.2.1. Limitations

The present study investigated the feasibility of MEP operant conditioning in a small number of individuals with MS. Thus, while the initial results support a therapeutic possibility of this approach for improving locomotion in people with MS (and potentially other CNS disorders), its wider applicability is yet to be confirmed through future clinical studies of larger scales.

## Figures and Tables

**Table 1:** 

Subj.	Age	Yrs	EDSS	ROM		Strength		FIM		25 ft time (s)	
				Pre	Post	Pre	Post	Pre	Post	Pre	Post
A	56	27	4.0	N	WNL	3−	5	6	6	13.46	10.54
				(WNL)	(WNL)	(5)	(5)				
B	57	35	5.0	WNL	N	1	3	6	6	26.45	19.51
				(N)	(10)	(0)	(1−)				
C	47	26	5.0	WNL	WNL	2	3	6	6	27.51	36.80^∗^
				(WNL)	(WNL)	(2)	(3)				
D	54	30	5.5	WNL	WNL	4+	5−	2	2	39.84	33.80
				(WNL)	(WNL)	(5)	(5)				

Yrs: years since the original diagnosis of MS; EDSS: Expanded Disability Status Scale; ROM: passive ankle dorsiflexion range of motion (WNL: within normal limits; N: to neutral); Strength: manual muscle strength testing score for dorsiflexion, graded from 0 to 5 (0: no contractions felt in the muscle; 5: normal). ROM and Strength were measured bilaterally. The first row in each subject is for the conditioned leg and the second row (assessments indicated in ()) is for the contralateral leg. ^∗^In subject C, the post functional assessment was performed one month after the completion of 24 conditioning sessions.
